# Accelerated Bone Loss in Transgenic Mice Expressing Constitutively Active TGF-β Receptor Type I

**DOI:** 10.3390/ijms241310797

**Published:** 2023-06-28

**Authors:** Parichart Toejing, Nithidol Sakunrangsit, Pinyada Pho-on, Chinnatam Phetkong, Asada Leelahavanichkul, Somyoth Sridurongrit, Matthew B. Greenblatt, Sutada Lotinun

**Affiliations:** 1 Center of Excellence in Skeletal Disorders and Enzyme Reaction Mechanism, Department of Physiology, Faculty of Dentistry, Chulalongkorn University, Bangkok 10330, Thailand; lookplanoi_@hotmail.com (P.T.); nithidol.s@hotmail.com (N.S.); pinyada.pph@gmail.com (P.P.-o.); phetkong.cp@gmail.com (C.P.); 2 Division of Immunology, Department of Microbiology, Faculty of Medicine, Chulalongkorn University, Bangkok 10330, Thailand; aleelahavanit@gmail.com; 3 Department of Anatomy, Faculty of Science, Mahidol University, Bangkok 10330, Thailand; somyoth.sri@mahidol.ac.th; 4 Department of Pathology and Laboratory Medicine, Weill Cornell Medicine and Research Division, Hospital for Special Surgery, New York, NY 10065, USA; mag3003@med.cornell.edu

**Keywords:** TGF-β, osteoblast, osteoclast, bone, hardness

## Abstract

Transforming growth factor beta (TGF-β) is a key factor mediating the intercellular crosstalk between the hematopoietic stem cells and their microenvironment. Here, we investigated the skeletal phenotype of transgenic mice expressing constitutively active TGF-β receptor type I under the control of Mx1-Cre (*Mx1;TβRI^CA^* mice). μCT analysis showed decreased cortical thickness, and cancellous bone volume in both femurs and mandibles. Histomorphometric analysis confirmed a decrease in cancellous bone volume due to increased osteoclast number and decreased osteoblast number. Primary osteoblasts showed decreased ALP and mineralization. Constitutive *TβRI* activation increased osteoclast differentiation. qPCR analysis showed that *Tnfsf11/Tnfrsf11b* ratio, *Ctsk*, *Sufu*, and *Csf1* were increased whereas *Runx2*, *Ptch1, and Ptch2* were decreased in *Mx1;TβRI^CA^* femurs. Interestingly, *Gli1, Wnt3a*, *Sp7, Alpl, Ptch1, Ptch2*, and *Shh* mRNA expression were reduced whereas *Tnfsf11/Tnfrsf11b* ratio was increased in *Mx1;TβRI^CA^* mandibles. Similarly, osteoclast-related genes were increased in *Mx1;TβRI^CA^* osteoclasts whereas osteoblast-related genes were reduced in *Mx1;TβRI^CA^* osteoblasts. Western blot analysis indicated that SMAD2 and SMAD3 phosphorylation was increased in *Mx1;TβRI^CA^* osteoblasts, and SMAD3 phosphorylation was increased in *Mx1;TβRI^CA^* osteoclasts. CTSK was increased while RUNX2 and PTCH1 was decreased in *Mx1;TβRI^CA^* mice. Microindentation analysis indicated decreased hardness in *Mx1;TβRI^CA^* mice. Our study indicated that *Mx1;TβRI^CA^* mice were osteopenic by increasing osteoclast number and decreasing osteoblast number, possibly by suppressing Hedgehog signaling pathways.

## 1. Introduction

Transforming growth factor-beta (TGF-β) is a multi-functional growth factor that regulates proliferation, differentiation, apoptosis, and migration of many cell types including skeletal cells [1]. Three different isoforms of TGF-β—TGF-β1, TGF-β2 and TGF-β3—have been identified in mammals. However, TGF-β1 is the most abundant and widely expressed isoform, especially in bone. It plays a crucial roles in bone development and homeostasis, stimulates matrix protein synthesis, and is responsible for bone formation and resorption [2]. TGF-β is secreted as an inactive precursor which contains the signal peptide, the latency-associated protein (LAP), comprising the so-called latent TGF-β complex. Upon cleavage of the LAP by proteases, the active TGF-β is released. TGF-β acts through a heteromeric receptor complex of two types of transmembrane serine/threonine kinase receptors, type I (TβRI) and type II receptors (TβRII), at the cell surface. Canonical TGF-β signaling occurs when ligands bind to TβRII and phosphorylate TβRI. Phosphorylated TβRI initiates intracellular signaling through subsequent phosphorylation of downstream SMAD2 and SMAD3. The phosphorylated SMAD2 and SMAD3 form a complex with SMAD4 and translocate into the nucleus to regulate target gene transcription. TGF-β ligands also use non-canonical (non-SMAD2/3) signaling pathways to regulate cellular functions. TGF-β ligands can exert signals through activation of MAPK pathway responses, including activation of the p38, JNK, and ERK pathways, phosphoinositide 3-kinase/Akt, Rho GTPase, Wnt, and Notch signaling. The effects of TGF-β on MAPK signaling include both SMAD-dependent and SMAD-independent pathways [1]. Bone cells express TGF-β1, TGF-β2, and TGF-β3, and TGF-β induces both positive and negative effects on bone cells. TGF-β1 increases bone formation by recruiting osteoblast progenitors and stimulating their proliferation and differentiation in the early stages. On the other hand, it blocks the later phases of differentiation and mineralization [3]. Inhibition of TGF-β with the 1D11 antibody clone resulted in increased bone mineral density, trabecular thickness, and bone volume while decreasing active osteoclasts in bone marrow [4]. A biphasic effect of TGF-β1 on osteoclasts has also been reported. TGF-β1 stimulated osteoclast development at low doses by enhancing the *Tnfsf11/Tnfrsf11b* (RANKL/OPG) ratio while high concentrations of TGF-β1 downregulated *Tnfsf11*, leading to a reduced *Tnfsf11/Tnfrsf11b* ratio and a decrease in the numbers of osteoclast precursors [5].

*Tgfb1*^−/−^*Rag2*^−/−^ mice had reduced cancellous bone density and osteoblast number [6]. Osteoblast-specific mutant *Tgfb1* transgenic mice exhibited abnormal remodeling of mandibular condylar subchondral bone, including cartilage degradation and excessive chondrocyte apoptosis [7]. High concentrations of active TGF-β1 in subchondral bone induced abnormal subchondral bone formation and degeneration of articular cartilage, leading to osteoarthritis [8]. On the other hand, *TβRII*^−/−^ nestin^+^ mesenchymal progenitors decreased osteoarthritis development in an anterior cruciate ligament transection model. Deletion of *TβRII* in limbs using Prx1-cre resulted in the defects in joint development and a failure of chondrocyte hypertrophy [9]. Transgenic mice expressing a truncated kinase-defective *TβRII*, which acted as a dominant loss-of-function mutation, developed kyphoscoliosis and cartilage disorganization resembling osteoarthritis [10]. Moreover, *Tgfb2*-null mice caused defects in the skull base and vertebrae with reduced ossification, and delayed development [11]. Taken together, the effects of TGF-β or its receptors very much depend on both the target cells and biological context. However, no previous studies had been reported on the effects of constitutive *TβRI* activation in hematopoietic stem-cells-derived osteoclasts and osteoblasts on bone turnover.

We examined the impact of constitutive *TβRI* activation on bone turnover in 9-week-old transgenic mice with inducible expression of a constitutively active *TβRI* in hematopoietic stem-cells-derived osteoclasts and osteoblasts (*Mx1;TβRI^CA^* mice). Serum calcium levels were decreased whereas serum parathyroid hormone (PTH) levels were increased in *Mx1;TβRI^CA^* mice. *Mx1;TβRI^CA^* mice were osteopenic due to increased osteoclast numbers and decreased osteoblast numbers in both long bones and mandibles. Constitutive *TβRI* activation decreased osteoblast and increased osteoclast differentiation. Osteoclast-related genes including the *Tnfsf11/Tnfrsf11b* ratio*, Ctsk*, *Sufu*, and *Csf1* were increased, whereas *Runx2*, *Ptch1*, and *Ptch2* osteoblast-related genes were decreased in femurs. In mandibles, qPCR analysis indicated that *Gli1, Wnt3a*, *Sp7, Alpl, Ptch1, Ptch2*, and *Shh* were decreased, but the *Tnfsf11/Tnfrsf11b* ratio was increased. In addition, osteoclast-related genes were increased in *Mx1;TβRI^CA^* osteoclasts, whereas osteoblast-related genes were reduced in *Mx1;TβRI^CA^* osteoblasts. pSMAD2/total SMAD2 and pSMAD3/total SMAD3 was enhanced in *Mx1;TβRI^CA^* osteoblasts, whereas pSMAD3/total SMAD3 was increased in *Mx1;TβRI^CA^* osteoclasts. CTSK protein levels were increased, while RUNX2 and PTCH1 protein levels were decreased in *Mx1;TβRI^CA^* mice. *Mx1;TβRI^CA^* mice displayed decreased bone hardness.

## 2. Results

### 2.1. Generation of Mx1;TβRI^CA^ Mice

**Figure 1:**
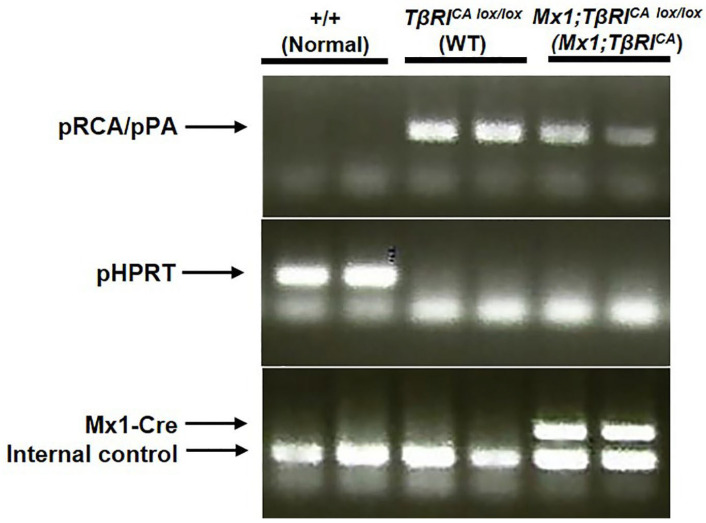
PCR of genomic DNA from mouse tails showing bands in Mx1;TβRI mice using pRCA/pPA, pHPRT, and Mx1-Cre primers.

*TβRI^CA lox/lox^* (*TβRI^CA^*) mice were crossed with mice expressing Cre recombinase under the control of the Mx1 promoter induced by a type I interferon following injection of polyinosinic-polycytidylic acid (poly (I:C) to generate *Mx1;TβRI^CA^* mice. Using pRCA/pPA primers, PCR analysis of tail DNA demonstrated lox/lox bands in WT and transgenic mice (Figure 1). As expected, WT (+/+) but not *TβRI^CA lox/lox^* mice showed a band using pHPRT primers, representing disruption of this locus via targeted insertion of the *TβRI^CA lox/lox^* transgene. However, Mx1-Cre primers detected bands only in *Mx1;TβRI^CA^* mice indicating effective constitutive expression of *TβRI* in hematopoietic stem-cells-derived osteoclasts and osteoblasts.

### 2.2. Mx1;TβRI^CA^ Mice Decreases Serum Calcium and Increases Serum PTH Levels

**Figure 2:**
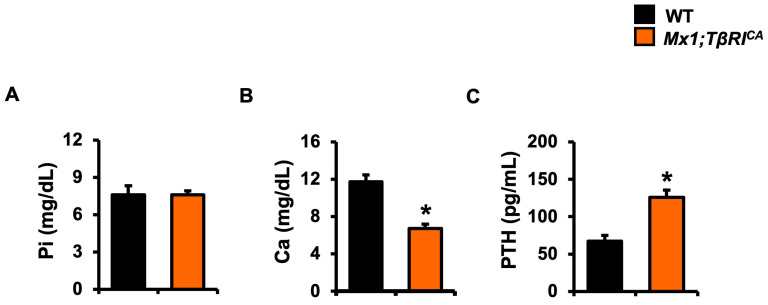
Serum chemistries of *Mx1;TβRI^CA^* mice. Serum levels of (**A**) phosphorus (*n* = 6), (**B**) calcium (*n* = 6), and (**C**) PTH (*n* = 6) from *Mx1;TβRI^CA^* mice compared to WT mice. Data are mean ± SEM. * *p* < 0.05 compared to WT.

To examine the changes in serum chemistries in *Mx1;TβRI^CA^* mice, serum phosphorus, calcium, and PTH were determined. There were no significant differences in serum phosphorus levels between the two groups (Figure 2A). Serum calcium levels were decreased while serum PTH levels were increased in *Mx1;TβRI^CA^* mice compare to WT controls (Figure 2B,C).

### 2.3. Mx1;TβRI^CA^ Mice Have Cancellous and Cortical Bone Loss in Femurs and Mandibles

**Figure 3:**
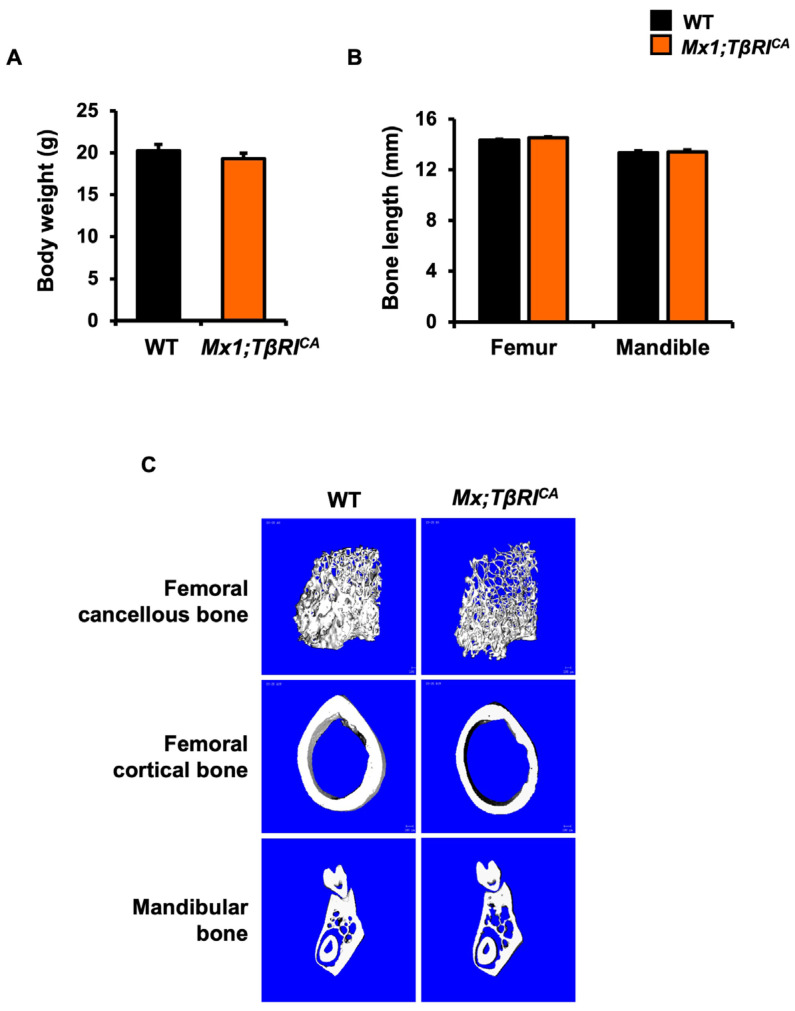

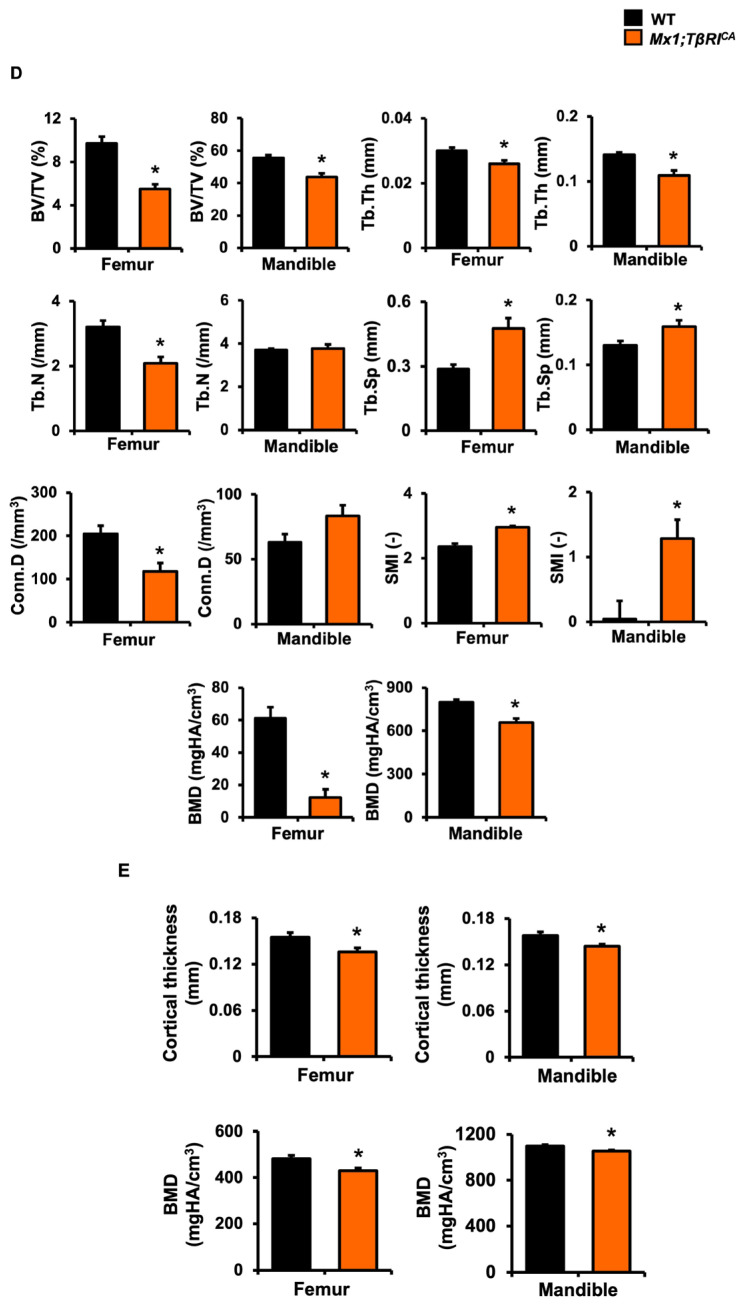
*Mx1;TβRI^CA^* mice are osteopenic. (**A**) Body weight (*n* = 6). (**B**) Femur and mandible length (*n* = 6). (**C**) Representative µCT images of femurs and mandibles. (**D**) µCT analysis of cancellous bone in femurs and mandibles from *Mx1;TβRI^CA^* mice compared to WT controls (*n* = 5–6). (**E**) µCT analysis of cortical bone in femurs and mandibles from *Mx1;TβRI^CA^* mice compared to WT controls (*n*= 5–6). Data are mean ± SEM. * *p* < 0.05 compared to WT. BV/TV; bone volume per tissue volume, Tb.Th; trabecular thickness, Tb.N; trabecular number, Tb.Sp; trabecular separation, Conn.D; connectivity density, SMI; structural model index, and BMD; bone mineral density.

There was no significant difference in body weight between *Mx1;TβRI^CA^* mice and WT mice (Figure 3A). Femur and mandible length was similar (Figure 3B). To evaluate whether the constitutive activation of *TβRI* impacted femoral and mandibular cancellous and cortical bone in 9-week-old mice, µCT analysis was performed. µCT images of femoral and mandibular bone in WT and *Mx1;TβRI^CA^* mice are shown in Figure 3C. *Mx1;TβRI^CA^* mice were osteopenic in both femurs and mandibles. Bone microstructural parameters are shown in Figure 3D. Both femurs and mandibles in *Mx1;TβRI^CA^* mice showed a significant decrease in cancellous bone volume, trabecular thickness, and bone mineral density with a concomitant increase in trabecular separation and structural model index compared to WT controls. Trabecular number and connectivity density was decreased in the femur whereas it did not change in mandibles. The skeletal phenotype in mandibles is milder than femurs. Cortical thickness and bone mineral density were significantly reduced in both the femurs and mandibles of *Mx1;TβRI^CA^* mice (Figure 3E). These findings demonstrated that femoral and mandibular bone loss was occurred in both cancellous and cortical bone in *Mx1;TβRI^CA^* mice.

### 2.4. Mx1;TβRI^CA^ Mice Had Decreased Bone Formation and Increased Bone Resorption

Similar to µCT data, histomorphometric analysis of tibiae and mandibles indicated cancellous bone loss. As shown in Table 1, *Mx1;TβRI^CA^* mice had decreased cancellous bone volume, trabecular thickness, trabecular number, and increased trabecular separation compared to WT mice. Furthermore, osteoblast surface per bone surface, osteoblast number per bone perimeter, and osteoblast number per tissue area were reduced in *Mx1;TβRI^CA^* mice. In addition, *TβRI^CA^* mice had increased osteoclast surface per bone surface, osteoclast number per bone perimeter, osteoclast number per tissue area, and eroded surface in *Mx1;TβRI^CA^* mice. Therefore, our data confirmed that *Mx1;TβRI^CA^* mice displayed altered bone turnover due to decreased bone formation and increased bone resorption leading to bone loss.

### 2.5. Mx1;TβRI^CA^ Mice Have Decreased Osteoblast Differentiation and Increased Osteoclast Differentiation

**Figure 4:**
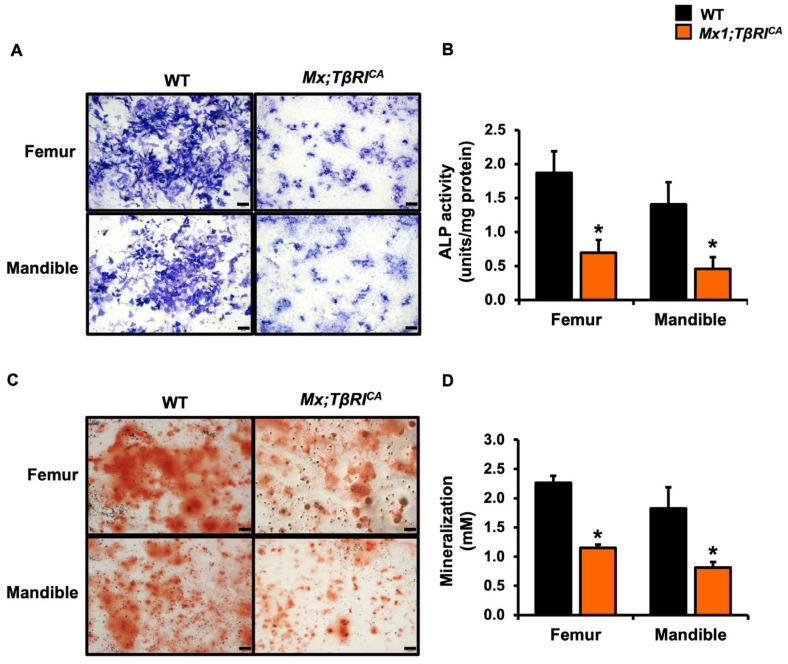
Constitutive activation of *TβRI* decreases osteoblast differentiation. (**A**) ALP staining in osteoblasts derived from the long bones and mandibles of *Mx1;TβRI^CA^* mice compared to WT controls. 4× Magnification, Scale bar = 200 μm. (**B**) ALP activity in osteoblasts (*n* = 4). (**C**) Alizarin red staining in osteoblasts derived from long bones and mandibles of *Mx1;TβRI^CA^* mice compared to WT controls. 4× Magnification, Scale bar = 200 μm. (**D**) Mineralization in osteoblasts (*n* = 5). Results are mean ± SEM. * *p* < 0.05 compared to WT.

Histomorphometry indicated that constitutive activation of *TβRI* led to a decrease in osteoblast number and increase in osteoclast number. To confirm that constitutive *TβRI* activation decreased osteoblast differentiation, we performed an in vitro assay using primary osteoblast progenitors derived from long bones and mandibles. ALP and mineralized bone nodules were decreased in osteoblasts derived from both *Mx1;TβRI^CA^* long bones and mandibles, indicating that exogenous mutated *TβRI* reduced osteoblast differentiation (Figure 4).

**Figure 5:**
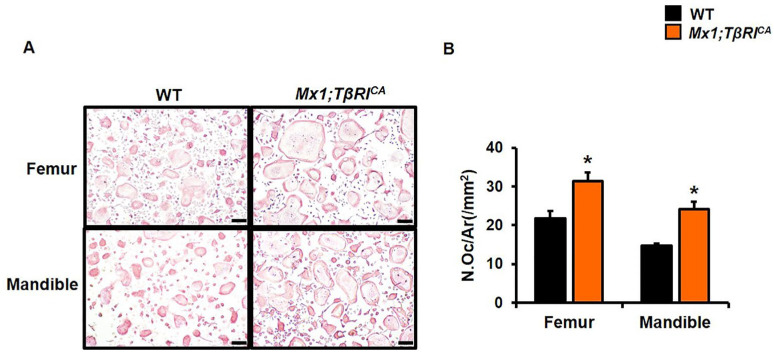
Constitutive activation of *TβRI* increases osteoclast differentiation. (**A**) TRAP staining in osteoclasts. 10x Magnification, Scale bar = 100 μm. (**B**) Osteoclast number per area (N.Oc/Ar) from long bones and mandibles in *Mx1;TβRI^CA^* mice compared to WT control (*n* = 4). Results are mean ± SEM. * *p* < 0.05 compared to WT controls.

We next determined whether *Mx1;TβRI^CA^* mice had increased osteoclast number. An in vitro osteoclast differentiation assay was performed using long bone and mandibular osteoclast precursors. Consistent with the increased bone resorption observed in vivo, constitutive *TβRI* activation increased TRAP positive osteoclasts derived from bone marrow macrophages (BMMs) of *Mx1;TβRI^CA^* mice compared to WT controls (Figure 5). These data revealed a major role of *TβRI* in osteoblast and osteoclast differentiation.

### 2.6. Mx1;TβRI^CA^ Mice Have Decreased Osteoblast and Increased Osteoclast-Related Gene Expression

**Figure 6:**
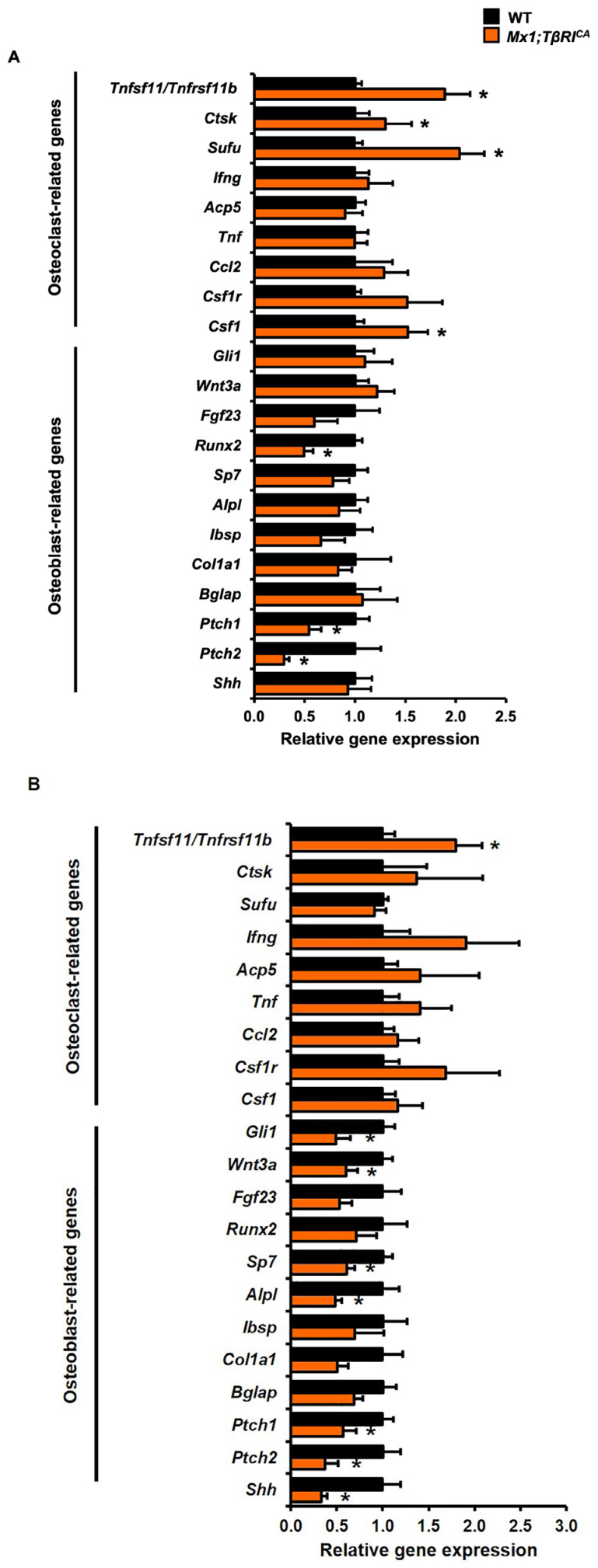
Constitutive *TβRI* activation increases osteoclast- and decreases osteoblast-related gene expression in femurs and mandibles. (**A**) qPCR analysis of osteoblast and osteoclast related genes expression in femurs (*n* = 5–6) and (**B**) mandibles from *Mx1;TβRI^CA^* mice compared to WT controls (*n* = 5–6). Results are mean ± SEM. * *p* < 0.05 compared to WT.

To investigate the mechanisms by which constitutive activation of *TβRI* decreased bone formation and increased bone resorption, qPCR was performed to determine the expression of osteoblast- and osteoclast-associated transcripts in femurs and mandibles. The expression of *Runx2*, a key transcription factor for osteoblast differentiation, was significantly reduced in *Mx1;TβRI^CA^* femurs compared to WT controls (Figure 6A). There was no significant difference in the expression of a number of genes influencing osteoblastogenesis, including *Gli1, Wnt3a, Fgf23, Sp7, Alpl, Ibsp, Col1a1, Bglap*, and *Shh*. For mandibular bones, the levels of osteoblast-related genes, *Sp7*, and *Alpl* were decreased in *Mx1;TβRI^CA^* mice (Figure 6B). *Fgf23, Runx2, Ibsp, Col1a1*, and *Bglap* were not altered, arguing that the reduction in *Runx2* expression in femurs observed reflected a specific downregulation of *Runx2* itself as opposed to reflecting a general paucity of osteoblasts. Hedgehog and Wnt signaling are essential regulators of osteoblast proliferation and differentiation, through the expression of *Gli1*, *Shh*, *Ptch1, Ptch2*, and *Wnt3a. Gli1*, *Shh*, and *Wnt3a* were reduced in *Mx1;TβRI^CA^* mandibles but not femurs. However, *Ptch1* and *Ptch2* were decreased in both *Mx1;TβRI^CA^* mandibles and femurs. Bone loss depends on the ratio of *Tnfsf11/Tnfrsf11b*, as this ratio is a major regulator of osteoclast differentiation and survival. *Tnfsf11/Tnfrsf11b* gene expression was significantly increased in both femurs and mandibles of *Mx1;TβRI^CA^* mice compared to WT controls (Figure 6A,B). Moreover, *Ctsk*, *Sufu*, and *Csf1*, regulators of bone resorption, were increased in femoral bone of *Mx1;TβRI^CA^* mice (Figure 6A). These findings demonstrated that *Mx1;TβRI^CA^* mice exhibited femoral and mandibular bone loss associated with reduced osteoblast- and increased osteoclast-related gene expression.

**Figure 7:**
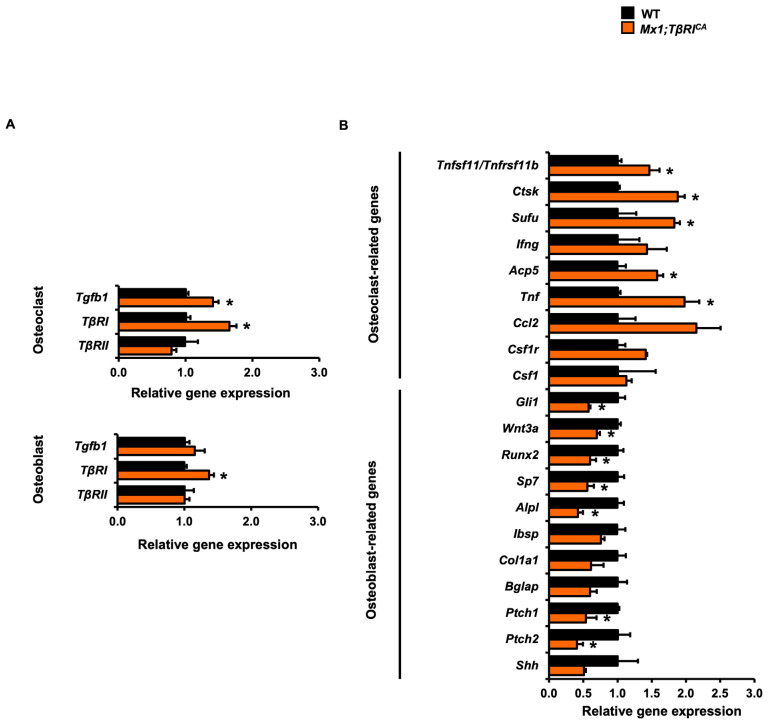
Constitutive *TβRI* activation increases osteoclast- and decreases osteoblast-related gene expression in vitro. (**A**) qPCR analysis of TGF-β and their receptors from osteoblasts and osteoclasts (*n* = 3). (**B**) osteoblast and osteoclast related genes expression in vitro derived from long bone of *Mx1;TβRI^CA^* mice compared to WT controls (*n* = 3–4). Results are mean ± SEM. * *p* < 0.05 compared to WT.

To confirm these gene expression changes observed in femurs and mandibles, osteoblasts and osteoclasts derived from long bones were studied to determine the expression of osteoblast- and osteoclast-related genes by qPCR analysis in vitro. The gene expression levels of *TβRI* significantly increased in *Mx1;TβRI^CA^* osteoblasts and osteoclasts compared to WT controls, confirming that *Mx1;TβRI^CA^* cells had an overexpression of *TβRI* as expected (Figure 7A)*. Tgfb1* expression significantly increased in osteoclasts but not osteoblasts. However, there was no difference in *TβRII* expression in osteoblasts and osteoclasts between *Mx1;TβRI^CA^* and WT mice. The expression of *Runx2*, a key transcription factors for osteoblast differentiation, was significantly reduced in *Mx1;TβRI^CA^* mice compared to WT controls (Figure 7B). *Sp7* and *Alpl* were decreased in *Mx1;TβRI^CA^* mice. However, expression of *Ibsp, Col1a1*, and *Bglap* was not altered. Interestingly, Hedgehog-related genes, *Gli1*, *Ptch1, Ptch2*, and Wnt signaling-related gene, *Wnt3a*, were decreased in *Mx1;TβRI^CA^* mice compared to WT controls (Figure 7B). *Tnfsf11/Tnfrsf11b* gene expression was significantly increased in *Mx1;TβRI^CA^* osteoclasts compared to WT controls (Figure 7B). Moreover, *Ctsk*, *Sufu*, *Acp5*, and *Tnf* were increased in *Mx1;TβRI^CA^* osteoclasts as well (Figure 7B). These results demonstrated that *Mx1;TβRI^CA^* mice exhibited bone loss associated with reduced osteoblast- and increased osteoclast-related gene expression.

### 2.7. Mx1;TβRI^CA^ Mice Have Increased TGF-β Signaling Leading to Decreases in RUNX2 and PTCH1 and Increases in CTSK Protein Levels

**Figure 8:**
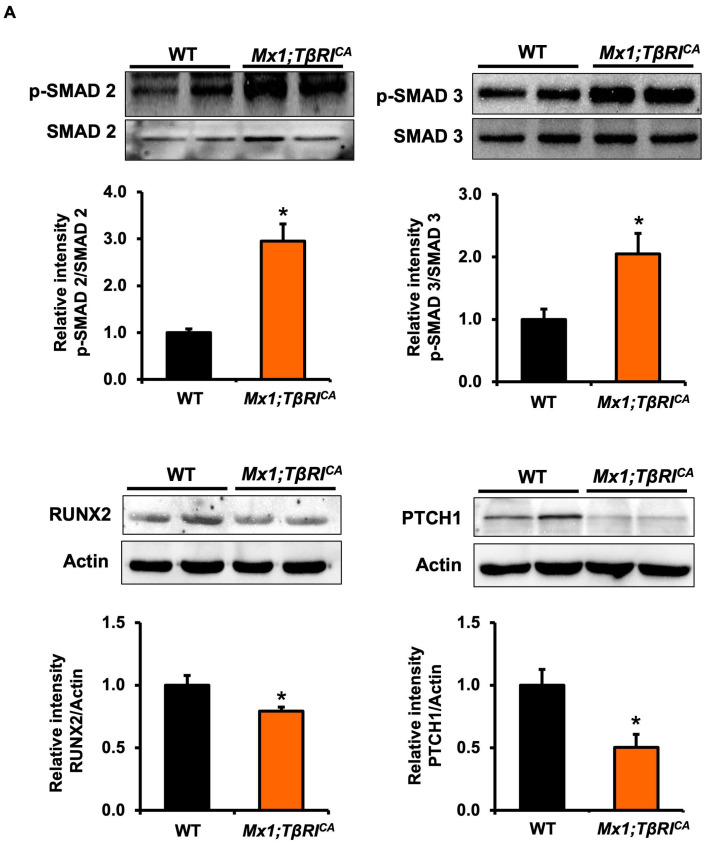

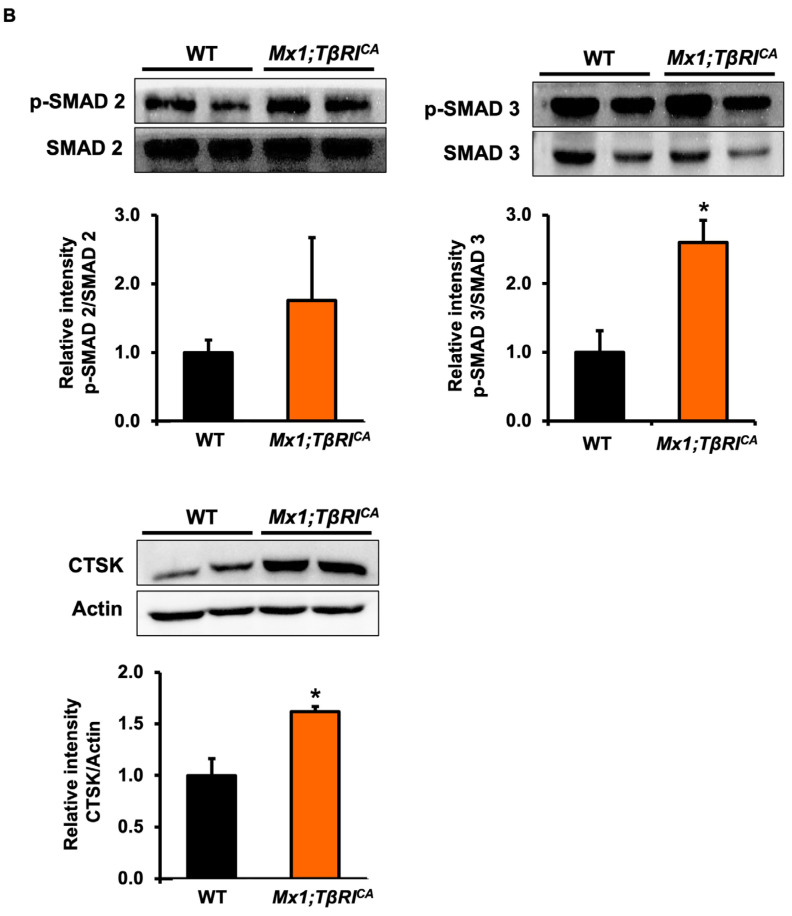
*Mx1;TβRI^CA^* mice induced TGF-β signaling, decreased RUNX2, and PTCH1 expression and increased CTSK expression. (**A**) SMAD2 and 3 phosphorylation, RUNX2 and PTCH1 protein levels in osteoblasts cells derived from long bones of *Mx1;TβRI^CA^* mice compared to WT controls (*n* = 4). (**B**) SMAD2 and 3 phosphorylation, CTSK protein levels in osteoclast cells derived from long bones of *Mx1;TβRI^CA^* mice compared to WT controls (*n* = 4). Results are mean ± SEM. * *p* < 0.05 compared to WT.

To explore the role of constitutive activation of *TβRI* on the TGF-β signaling pathway, Western blot analysis for phosphorylated SMAD 2 and 3 (pSMAD 2 and 3) was performed alongside examining the expression of osteoblast and osteoclast-specific proteins. We found that the ratio of pSMAD2/total SMAD 2 and pSMAD3/total SMAD 3 was significantly increased in osteoblasts from *Mx1;TβRI^CA^* mice compared to WT controls (Figure 8A). Constitutive activation of *TβRI* increased the ratio of pSMAD3/total SMAD3 but not pSMAD2/total SMAD2 in osteoclasts (Figure 8B). In addition, a key transcription factor for osteoblast formation, RUNX2 protein levels, were significantly reduced in *Mx1;TβRI^CA^* mice compared to WT control (Figure 8A). Consistently, levels of PTCH1, transmembrane protein receptor of Hedgehog signaling pathway, were decreased (Figure 8A). Furthermore, levels of CTSK, an osteoclast-specific protein, were increased in *Mx1;TβRI^CA^* mice (Figure 8B). These results confirmed that constitutive activation of *TβRI* cause bone loss by affecting osteoblast and osteoclast protein levels.

### 2.8. Mx1;TβRI^CA^ Mice Have Decreased Bone Hardness in Tibia

**Figure 9:**
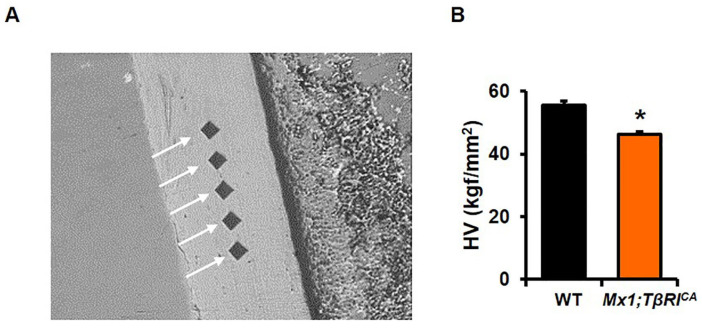
*Mx1;TβRI^CA^* mice have decreased bone hardness. (**A**) Five indents of tibial cortical mid-shaft. (**B**) Hardness of tibial cortical bone in *Mx1;TβRI^CA^* mice compared to WT controls (*n* = 8). Results are mean ± SEM. * *p* < 0.05 compared to WT. HV; Vickers hardness.

*Mx1;TβRI^CA^* mice display bone loss. To determine whether, in addition to a reduction in bone volume, the mechanical properties of the cortical bone are compromised, we performed microindentation hardness testing of the tibial midshaft. The tibial cortical bone was tested with five indents (Figure 9A) and the hardness was determined. *Mx1;TβRI^CA^* mice displayed significantly reduced cortical bone hardness compared to WT mice (Figure 9B).

## 3. Discussion

TGF-β, a pleiotropic growth factor, binds to TβRII and then activates TβRI leading to induction of downstream signaling pathways and ultimately regulation of bone metabolism [1]. It was previously demonstrated that *TβRI* and *TβRII* are expressed on osteoblasts and osteoclasts [12]. Although the effects of *TβRII* on bone and cartilage homeostasis are well documented, the function of *TβRI* in bone function was poorly defined. Here, we generated *Mx1;TβRI^CA^* mice in which *TβRI* was conditionally activated in hematopoietic stem-cells-derived osteoclasts and osteoblasts in order to determine the physiological role of TGF-β in skeletogenesis. Mx1-Cre is silent in normal mice but can be induced in interferon-responsive cells, including monocytes and macrophages, following administration of poly (I:C) [13,14]. We confirmed the genotype of study mice by PCR, demonstrating that mice showed the Mx1-Cre band after poly (I:C) injection to induce TβRI expression. Hematopoietic stem cells are capable of differentiating into macrophages and osteoclasts [15]. In this study, we found that gene expression and protein levels of osteoclast markers were increased in *Mx1;TβRI^CA^* mice. These increased osteoclast markers indicated that the overexpression of *TβRI* in hematopoietic stem-cells-derived osteoclasts and osteoblasts caused an increase in the differentiation of hematopoietic stem cells to osteoclasts. Likewise, previous evidence found that TGF-β stimulates the formation of osteoclast from hematopoietic stem cells [16].

Our results showed that *Mx1;TβRI^CA^* mice were osteopenic through increased osteoclast-mediated bone resorption and decreased osteoblastic bone formation. Furthermore, osteoblast formation was decreased by suppressing Hedgehog signaling pathways. As expected, we found that calcium levels were lower in *Mx1;TβRI^CA^* mice than WT controls. Abnormalities of calcium and phosphate metabolism can in turn impact secretion of PTH. Interestingly, the present study observed that serum PTH levels were increased in *Mx1;TβRI^CA^* mice. High levels of PTH can induce osteoclastic bone resorption [17]. The PTH receptor is expressed on osteoblasts and activates the PKA pathway, leading to increased secretion of *Tnfsf11* [18]. Administration of PTH in male rat increased the levels of TGF-β1 [19]. PTH enhanced TGF-β1 expression and secretion through the PKC pathway and increased TGF-β2 expression and secretion through the PKA pathway [20]. These findings may indicate that the increased PTH levels in *Mx1;TβRI^CA^* mice are due to low serum calcium levels, thus increasing bone resorption.

μCT analysis indicated that cancellous bone volume, trabecular thickness, cortical thickness, and BMD in both femurs and mandibles were significantly decreased in *Mx1;TβRI^CA^* mice, indicating the presence of osteopenia. Several studies have reported that increasing TGF-β signaling results in bone defects. TGF-β stimulated signaling via formation of heteromeric complexes of type I and type II serine/threonine kinase receptors and initiated intracellular signaling through both SMAD-dependent and SMAD-independent signaling pathways. Overexpression of *TβRII-B*, a spliced form of *TβRII*, in chondrocytes was associated with increases in SMAD2 phosphorylation, SMAD3 signaling, and type II collagen production [21]. Overexpression of *Tgfb1* caused abnormal bone remodeling and mandibular condylar cartilage degradation in osteoblast-specific *Tgfb1* mutants [7]. Osteoblast-specific overexpression of *Tgfb2* using *Ocn* promoter caused a mineralization defect and severe hypoplasia of clavicles similar to cleidocranial dysplasia [22]. This present study found that the constitutive activation of *TβRI* in mice resulted in osteopenia.

Bone homeostasis is maintained by the process of bone formation by osteoblast and bone resorption by osteoclast. Histomorphometric analysis indicated that cancellous bone volume was decreased in *Mx;TβRI^CA^* tibiae and mandibles compared to WT controls. Osteoblast numbers in tibial and mandibular bone in *Mx;TβRI^CA^* mice were reduced. Microindentation testing revealed that *Mx1;TβRI^CA^* mice had decreased bone hardness consistent with osteopenia from μCT and histomorphometric analysis. The TβRI kinase inhibitor, SD-208, increased bone mass by increasing osteoblast differentiation and suppressing osteoclast differentiation [23]. Lian et al. demonstrated that TGF-β inhibited osteoblast differentiation by suppressing ATF4-dependent *Ocn* transcription [24]. TGF-β stimulates the early stages of osteoblast differentiation but inhibited later stages of osteoblast maturation and mineralization [3,25]. Hematopoietic stem cells serve as the source of osteoclast precursors. Mandibular and long bones of *Mx1;TβRI^CA^* mice had increased osteoclast numbers and differentiation status compared to those of WT controls. A previous study reported that TGF-β1 stimulated osteoclast maturation in fetal mouse long bones [26]. TGF-β1 exerted dual effects on hematopoietic stem cell proliferation depending on concentration [27]. High concentrations of TGF-β1 induced phosphorylation of SMAD3 and activated c-Jun and ATF-2. In contrast, low TGF-β1 concentrations activated STAT signaling through an SMAD3-independent pathway. Moreover, TGF-β induced the activation of an SMAD2/3-associated TRAF6-TAB1-TAK1 complex leading to stimulation of osteoclastogenesis in response to *Tnfsf11* stimulation [28]. *TβRI* deficiency using Dermo1-cre has been shown to have shortened long bones, decreased cancellous bone and bone collar mineralization, produced abnormalities in the perichondrium, and reduced osteoblast proliferation and differentiation [29]. Our in vitro assay indicated that constitutive activation of *TβRI* decreased osteoblast and increased osteoclast differentiation. *Mx1;TβRI^CA^* mice had decreased ALP activity and mineralization in long bones and mandibles compare to WT controls. In addition, the numbers of TRAP positive osteoclasts in both long bones and mandibles of *Mx1;TβRI^CA^* mice were increased. Therefore, the present study is the first to report that gain of function of *TβRI* decreased osteoblast numbers and differentiation together with increasing osteoclast numbers and differentiation, ultimately leading to bone loss.

Constitutive *TβRI* activation promoted osteoclast differentiation and formation by controlling genes involved in tumor necrosis factor (TNF) family signaling including *Tnfsf11* and *Tnfrsf11b*. Upregulation of the ratio of *Tnfsf11/Tnfrsf11b* expression in femurs and mandibles and apparent increases in osteoclasts derived from long bones were observed. *Tnfsf11* is synthesized and secreted from osteoblast-lineage cells and binds with RANK on osteoclasts, leading to increases in osteoclast formation, function, and survival. In addition, osteoblasts also secrete *Tnfrsf11b*, which acts as a soluble *Tnfsf11* decoy receptor for preventing *Tnfsf11* binding to RANK, thereby inhibiting osteoclast development [30]. Our result correlated with previous findings that increased TGF-β1 stimulation increased *Tnfsf11* gene expression in primary human osteoblasts cell culture via SMAD2/3 stimulation and that the *TβRI* inhibitor, SB431542, blocked these effects [31]. In addition, low concentrations of TGF-β increased the *Tnfsf11/Tnfrsf1b* ratio and *Csf1* expression, leading to induce osteoclastogenesis [5]. A previous study revealed that after *Tnfsf11* binds with its receptor, RANK, on osteoclasts, it leads to the activation of TRAF6, ERK, JNK, and p38, ultimately resulting in osteoclast proliferation and differentiation [32]. *Csf1* expression was increased in femoral bone of *Mx1;TβRI^CA^* compared to WT controls. Moreover, *Csf1* bound to *Csf1r* and stimulated PI3K/Akt and ERK, resulting in osteoclast proliferation and survival [32]. In addition, our qPCR analysis in femoral bone indicated that the expression of specific osteoclastogenesis-associated genes, including *Ctsk* and *Sufu*, was significantly increased in *Mx1;TβRI^CA^* mice. In the process of bone resorption, osteoclasts utilize cathepsin K, a lysosomal cysteine protease, to degrade collagen and other matrix proteins [33]. CTSK protein was also increased in *Mx1;TβRI^CA^* osteoclasts. The expression of *Ctsk* is regulated by *Tnfsf11* signaling and *Tnfsf11* activates the transcriptional factor, *Nfatc1*, to mediate stimulation of *Ctsk* gene expression in osteoclasts [34]. *Sufu*, a cytoplasmic protein, is a negative regulator of Hedgehog signaling pathway that binds to GLI family protein and suppress the differentiation of early skeletal progenitors. Deletion of *Gli2* and *Sufu* in the cranial neural crest enhanced calvarial bone formation [35]. Osteoporotic mice overexpressed *Sufu* [36]. *Sufu* deletion suppressed *Tnfsf11*-induced TRAP positive osteoclast differentiation, osteoclast gene expression, and osteoclast activity in vitro [37]. In addition, *Acp5 and Tnf* significantly increased in *Mx1;TβRI^CA^* osteoclasts but not in femurs and mandibles. Femurs and mandibles are tissues that have multiple factors and signaling networks. Thus, those factors may have been implicated in the levels of these genes. In addition, there are many cell types in femurs and mandibles. Therefore, our findings indicated that *Mx1;TβRI^CA^* mice displayed an increase in the expression of several pathways regulating osteoclast differentiation, leading to increases in osteoclast formation and subsequently increasing bone resorption.

*Wnt3a* expression was significantly reduced in *Mx1;TβRI^CA^* mice. *Wnt3a* activates the β-catenin phosphorylation via the canonical Wnt signaling pathway and then stimulates the downstream target of osteoblast-related genes, leading to osteoblast differentiation and bone formation [38]. *Runx2* is a DNA binding transcription factor that plays a key role in bone formation [39]. Interestingly, we found a significant reduction in *Runx2* gene and protein levels in *Mx1;TβRI^CA^* osteoblasts compared to WT controls. As expected, we demonstrated that levels of osteoblast-related genes, *Sp7* and *Alpl*, in *Mx1;TβRI^CA^* mandibles were significantly lower than those in WT controls. TGF-β activates ERK1/2 and JNK, which in turn leads to negatively regulate SMAD3-induced ALP activity and mineralization in MC3T3-E1 cells [40]. The Hedgehog signaling pathway has an essential role for the differentiation and development of osteoblasts. The multitransmembrane protein *Ptch1* and *Ptch2* are the receptors for Hedgehog while also being Hedgehog target genes. *Ptch1* and *Ptch2* expression was dramatically decreased in *Mx1;TβRI^CA^* femurs and mandibles whereas *Gli1* and *Shh* expression was reduced in *Mx1;TβRI^CA^* mandibles. PTCH1 protein levels also decreased in *Mx1;TβRI^CA^* osteoblasts by western blot analysis. *Shh* binds with *Ptch* receptors, leading to release and conformational change of *Smo* and activates transcriptional factors *Gli1*, 2 and 3 [41]. *Gli* translocates into the nucleus to induce transcription of Hedgehog target genes in osteoblasts. In addition, a reduction in bone mass caused by decreased osteoblast differentiation, and increased osteoclastogenesis were found in *Gli1*-haploinsufficient mice [42]. These findings suggest a crosstalk between TGF-β and Hedgehog signaling in *TβRI* activation as one of the mechanistic insights resulting from study of constitutive *TβRI* activation-induced bone loss. Furthermore, our study demonstrated that *Mx1;TβRI^CA^* mice induced SMAD 3 phosphorylation in osteoclasts, demonstrating the *TβRI^CA^* transgene was active. A previous study found that excessive TGF-β signaling increased SMAD 2 phosphorylation, leading to bone loss; conversely, the same study reported that inhibiting TGF-β signaling using the 1D11 neutralizing antibody improved the bone phenotype [43]. After *Tnfsf11* binds with RANK, this leads to the activation of Smad 2/3 to form the complex with TRAF6-TAB1-TAK1 that causes increased osteoclast differentiation [44].

**Figure 10:**
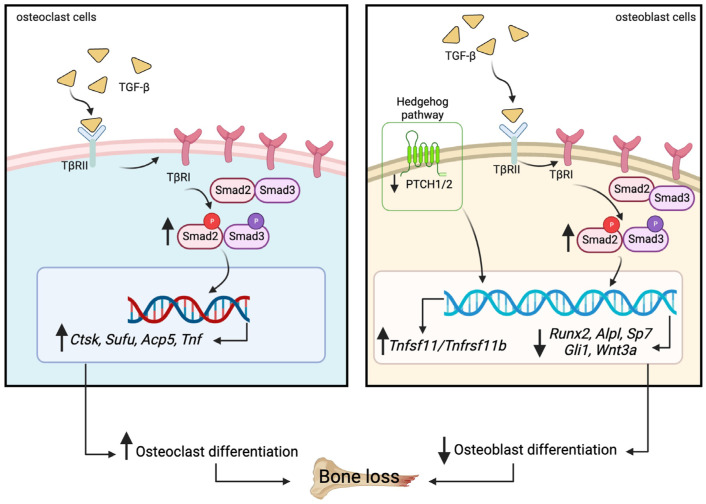
Constitutive activation of *TβRI* engages TGF-β signaling and affects osteoblast and osteoclast transcriptional profiles, leading to bone loss.

In addition, expression of *Tgfb1* and TβRI was increased in osteoclast cells, confirming overexpression of *TβRI*. However, previous studies found that mesenchymal stromal cells (MSCs) identified as a group of heterogeneous cells, comprising multipotent stem cells [45]. Bone-marrow MSCs are targeted by Mx1-cre upon induction [46,47]. In addition, the interferon-inducible Mx1 promoter drives the activation of Cre after administration of poly (I:C). Therefore, our results from primary osteoblasts found the increased *TβRI* gene expression and the ratio of pSMAD2/total SMAD2 and pSMAD3/total SMAD3 in osteoblasts. Previous studies reported that TGF-β stimulates the early stages of osteoblast differentiation but inhibits later stages of osteoblast differentiation [3,25]. Interestingly, this may explain the reduced osteoblast numbers seen in *Mx1;TβRI^CA^* mice in our study. Taken together, our results indicated that *Mx1;TβRI^CA^* mice displayed decreased osteoblast formation associated with reductions in several osteoblast-related genes, including *Wnt3a*, *Runx2*, *Sp7*, *Alpl*, and Hedgehog target genes (Figure 10).

In summary, constitutive *TβRI* activation induces osteopenia by both increasing osteoclast differentiation and decreasing osteoblast differentiation, possibly through the suppression of the Hedgehog signaling pathway. Serum calcium were decreased whereas serum PTH was increased. Nowadays, there are many pharmacologic treatments for osteoporosis such as bisphosphonates, estrogen-related therapies, parathyroid hormone analogs, and RANK-ligand inhibitory antibodies [48]. However, our study revealed that constitutive *TβRI* activation is able to produce an osteopenic phenotype. Inhibitors of TGF-β and their receptors are currently under clinical development in phase I/II trials, especially in cancer treatment [49]. Thus, this study indicates that the bone mass effects of these drugs should be monitored for possible increases. Moreover, TβRI inhibitors may represent an adjuvant therapy for the treatment of osteoporosis or other disorders of low bone mass.

## 4. Materials and Methods

### 4.1. Mice

Mice from Dr. Laurent Bartholin were transferred from the Department of Anatomy, Faculty of Science, Mahidol University, Bangkok, Thailand. *TβRI^CA^* mice were prepared according to the methods described by Vincent et al. [50]. Briefly, constitutively active *TβRI* was knocked into the X chromosome-linked hypoxanthine phosphoribosyl transferase (HPRT) locus for generating *TβRI^CA^* mice. Therefore, female mice were used in this study. *TβRI^CA^* mice were crossed with mice expressing Cre recombinase under the control of the interferon-inducible Mx1 promoter to generate *Mx1;TβRI^CA^* mice to target hematopoietic stem cells. The genotype of 3-week-old female mice was determined by PCR analysis of their tail DNA. Genotyping PCR was performed using 3 primers, pRCA/pPA, pHPRT-f/pHPRT-r, and Mx1-Cre/Internal control.

pRCA: 5′-TTG TGA ACA GAA GTT AAG GC-3′
pPA: 5′-AGA AAG AAC AAT CAA GGG TCC-3′
pHPRT-f: 5′-GAG GGA GAA AAA TGC GGA GTG-3′
pHPRT-r: 5′-CTC CGG AAA GCA GTG AGG TAA G-3′
Mx1-Cre-f: 5′-GTG AGT TTC GTT TCT GAG CTC C-3′
Mx1-Cre-r: 5′-CGG TTA TTC AAC TTG CAC CA-3′
Internal control-f: 5′-GAC AAA ATG GTG AAG GTC GG-3′
Internal control-r: 5′-CAA AGG CGG AGT TAC CAG AG-3′

*Mx1;TβRI^CA^* mice were injected intraperitoneally with 1 mg/mL of poly (I:C) in PBS. Two genotypes; *TβRI^CA lox/lox^* as a WT control and *Mx1;TβRI^CA lox/lox^* (*Mx1;TβRI^CA^*) as mice that overexpressed *TβRI* were examined. Mice were housed at the Faculty of Medicine, Chulalongkorn University under controlled temperature (25 ± 2 °C) and with a 12 h light/dark cycle and free access to standard rodent chow (C.P. Mice Feed, Perfect Companion Group Co., Ltd., Bangkok, Thailand) and water. The animal protocol was approved by the Institutional Animal Care and Use Committee (IACUC) at the Faculty of Medicine, Chulalongkorn University and conducted according to the guidelines of the Animal Research: Reporting In Vivo Experiments (ARRIVE, Singapore). Nine-week-old female *Mx1-cre;TβRI^CA^* mice and their WT controls were used. At the end of the experiment, mice were anesthetized with isoflurane. Blood samples were collected and serum was kept at −80 °C for determination of serum chemistries. The left mandibles, femurs, and tibiae were removed and fixed in 70% alcohol for μCT analysis, histomorphometry, and microindentation analysis. The right mandibles and femurs were frozen in liquid nitrogen and kept at −80 °C for RNA isolation and qPCR analysis.

### 4.2. µCT Analysis

μCT analysis was performed using a desktop μCT35, (Scanco Medical AG, Bassersdorf, Switzerland) in accordance with suggested guidelines [51]. The scan was subjected to Gaussian filtration and segmentation using a voltage of 70 kV, 113 μA, 12 μm and 7 μm voxel size for mandibles and femurs, respectively, and 800 ms integration time at a threshold of 190, and 350 for cancellous and cortical bone, respectively. Bone volume (BV/TV, %), trabecular thickness (Tb.Th, mm), trabecular number (Tb.N, /mm), trabecular separation (Tb.Sp, mm), connectivity density (Conn.D /mm^3^), structural model index (SMI, -), cortical thickness (mm), and bone mineral density (BMD, mgHA/cm^3^) were analyzed.

### 4.3. qPCR Analysis

Femur metaphysis and whole mandibles were harvested and snap-frozen in liquid nitrogen. The bones were placed in a pre-cooled mortar and grounded into fine powder using a pestle with liquid nitrogen. RNA was extracted using TRIzol (Invitrogen, Waltham, MA, USA), followed by RNA clean up using an RNeasy Mini kit (Qiagen, Germantown, MD, USA). The RNA yields were then determined using a NanoDrop 1000 (Thermo Fisher Scientific, Waltham, MA, USA). First-strand cDNA was synthesized using 1 μg of total RNA with SuperScript VILO cDNA synthesis kit (Invitrogen, Carlsbad, CA, USA) for reverse transcription. The qPCR was performed in Luna Universal qPCR master mix (New England Biolabs, Ipswich, MA, USA) using CFX96™ Optics Module (Bio-Rad, Hercules, CA, USA). The qPCR was conducted at 57 °C for 39 cycles. Gene expression signals were normalized to *gapdh* expression. All primer sequences are listed in Supplementary Table S1.

### 4.4. Osteoclast Culture

Briefly, bone marrow was flushed from mandibles and long bones using a needle (25 G) and with α-MEM medium. Bone marrow cells were passed through a 40 μm filter. Cells were then cultured in α-MEM medium containing 10% FBS, 100 units/mL penicillin, and 100 ug/mL streptomycin overnight. Nonadherent cells were placed on coverslips in the same medium containing 20 ng/mL M-CSF (R&D Systems, Inc., Minneapolis, MN, USA) for 2 days to generate BMMs. After that, BMMs were cultured in α-MEM medium containing 20 ng/mL M-CSF, and 3.3 ng/mL RANKL (R&D Systems, Inc., Minneapolis, MN, USA) for 6 days. TRAP-positive osteoclast number per total area (N.Oc/Ar) was quantified using OsteoMeasure software version 4.2.0.1 (Decatur, GA, USA).

### 4.5. Osteoblast Culture

The isolation of primary osteoblasts was prepared according to the methods described by Chevalier et al. [52]. After flushing the bone marrow out from mandibles and long bones, bones were minced with sterile scissors into small pieces and placed in α-MEM medium containing 1 mg/mL collagenase type II (Worthington Biochemical Corporation, Lakewood, NJ, USA) for 2 h at 37 °C with shaking. Then, cells were centrifuged at 5000 rpm for 8 min, and the supernatant was removed. Bone fragments were transferred to 75 cm^2^ flasks containing α-MEM, 20% FBS, 100 units/mL penicillin, and 100 μg/mL streptomycin until cells were confluent. Cells were cultured in α-MEM medium containing 20% FBS, 5 mM β-glycerophosphate, 50 μg/mL ascorbic acid, and 10 μM dexamethasone for 7 and 25 days. Osteoblasts were fixed with 3.7% formaldehyde and stained with Fast Blue RR (Sigma, St. Louis, MO, USA) for ALP on day 7. For mineralized bone nodules, osteoblasts were stained with 2% alizarin red (Sigma, St. Louis, MO, USA) on day 25. Mineralization was determined by destained alizarin red using 10% cetylpyridinium chloride in 10 mM sodium phosphate and concentration of alizarin red (mM) was quantified.

### 4.6. Histomorphometry

Mandibles were decalcified in 10% EDTA and 0.05 M Tris buffer, pH 7.3 for 3 weeks. Samples were dehydrated with ascending graded alcohol, xylene, and embedded in paraffin. Mandibles were sectioned on a Leica RM2255 microtome (Leica Biosystems Nussloch GmbH, Nußloch, Germany) at 5 μm thickness. Samples were deparaffinized and stained with hematoxylin and eosin. For tibiae, samples were processed undecalcified and embedded in methyl methacrylate. Osteoblast and osteoclast were counted for determining osteoblast surface per bone surface (Ob.S/BS, %), osteoblast number per bone perimeter (N.Ob/B.Pm,/mm), osteoblast number per tissue area (N.Ob/T.Ar, mm^2^), osteoclast surface per bone surface (Oc.S/BS, %), osteoclast number per bone perimeter (N.Oc/B.Pm, /mm), osteoclast number per tissue area (N.Oc/T.Ar, /mm^2^), and eroded surface per bone surface (ES/BS, %). Cancellous bone volume per tissue volume (BV/TV, %), trabecular thickness (Tb.Th, μm), trabecular separation (Tb.Sp, μm), and trabecular number (Tb.N,/mm) were assessed. All histomorphometric analysis was carried out using the OsteoMeasure system (OsteoMetric Inc.) according to standardized nomenclature [53].

### 4.7. Western Blot Analysis

Osteoblasts and osteoclasts were harvested and lysed using RIPA lysis buffer (Abcam, Cambridge, UK). Sample concentration was measured by BCA protein assay (ThermoFisher Scientific, Waltham, MA, USA). First, 20 ug of protein and a molecular weight marker (Bio-Rad, CA, USA) were loaded onto 10% SDS-PAGE gel and transferred to a PVDF membrane. Membranes were blocked with 5% non-fat dry milk in 0.1% Tween-20 (TBST) at room temperature for 1 h. After blocking, membranes were incubated with primary antibodies for RUNX2 (1:750, ab76956), Patched/PTCH1 (1:1000, ab53715), CTSK (1:1000, ab187647), SMAD2 (1:1000, ab33875), SMAD2 (phospho S465) (1:1000, ab 216482), SMAD3 (1:1000, ab40854), and SMAD3 (phospho S423 + S425) (1:1000, ab52903), actin (1:1000, ab8226), at 4 °C overnight. Membranes were incubated with secondary antibody conjugated-horseradish peroxidase (HRP) (Goat-Anti-Rabbit, 1:5000, ab205718 or Goat-Anti-Mouse, 1:5000, ab205719) for 1 h at room temperature. An enhanced chemiluminescence (ECL) kit (Bio-Rad, CA, USA) was used to visualize protein bands. The relative expression of the target protein was calculated by the ratio of protein band to the β-actin band.

### 4.8. Microindentation Analysis

Tibiae were embedded without demineralization in methyl methacrylate. Tibiae were polished with 600, 800, 1200 grit abrasive sandpaper followed by 0.5 µm de-agglomerated alumina powder. The hardness of tibial cortical bone was determined using microhardness tester, FM-810 (Future-Tech Corp., Kawasaki, Japan). A Vickers diamond indenter with an applied load of 25 gf for 10 s was used. The cortical bone was tested at midshaft for five indents each 50 µm apart. The Vickers hardness equation was 1.854 L/D^2^. The average length of two diagonals (D) was measured in millimeter and the load (L) was input kilograms.

### 4.9. Measurement of Bone Length

Bone length was determined using vernier calipers, measuring from the proximal to distal ends of femurs and the most anterior point of the dentary to the most posterior point of the articular condyle for mandibles [54,55].

### 4.10. Serum Chemistry

Levels of serum phosphorus and calcium were measured according to the manufacturer’s instructions (Standbio Laboratory, Boerne, TX, USA). Serum PTH levels were determined using an ELISA kit (Quidel, San Diego, CA, USA).

### 4.11. Statistically Analysis

All statistical analyses were performed using SPSS 24 (IBM, Armonk, NY, USA). Independent *t*-test was used to compare the differences between two groups. Data are expressed as mean ± SEM. *p* < 0.05 were considered statistically significant.

## Figures and Tables

**Table 1:** Tibial and mandibular bone histomorphometric data of *Mx1; TβRI^CA^* mice and WT controls.

Parameters	Tibia		Mandible	
	WT (*n* = 8)	*Mx1;TβRI^CA^* (*n* = 7)	WT (*n* = 6)	*Mx1;TβRI^CA^* (*n* = 6)
BV/TV (%)	5.95 ± 0.62	2.44 ± 0.43 *	58.73 ± 3.10	33.93 ± 3.07 *
Tb.Th (μm)	35.41 ± 2.04	28.92 ± 1.67 *	66.74 ± 4.89	48.45 ± 3.72 *
Tb.N (/mm)	1.69 ± 0.18	0.84 ± 0.14 *	9.04 ± 0.84	7.01 ± 0.31 *
Tb.Sp (μm)	613.02 ± 82.29	1350.27 ± 209.88 *	47.77 ± 5.85	95.81 ± 7.77 *
Ob.S/BS (%)	21.38 ± 2.02	13.59 ± 1.48 *	6.59 ± 0.79	4.40 ± 0.39 *
N.Ob/T.Ar (/mm^2^)	69.42 ± 7.57	22.64 ± 4.61 *	120.11 ± 7.83	73.27 ± 4.32 *
N.Ob/B.Pm (/mm)	20.75 ± 1.96	13.25 ± 1.51 *	6.80 ± 0.52	5.26 ± 0.34 *
Oc.S/BS (%)	0.37 ± 0.05	1.63 ± 0.30 *	0.10 ± 0.02	0.60 ± 0.03 *
N.Oc/T.Ar (/mm^2^)	1.12 ± 0.17	2.39 ± 0.58 *	1.54 ± 0.37	5.68 ± 0.50 *
N.Oc/B.Pm (/mm)	0.33 ± 0.03	1.49 ± 0.28 *	0.08 ± 0.02	0.41 ± 0.04 *
ES/BS (%)	0.73 ± 0.08	2.55 ± 0.39 *	0.18 ± 0.06	0.98 ± 0.06 *

Results are mean ± SEM. * *p* < 0.05 compared to WT.

## Data Availability

All data are included in this article and its Supplementary Information File.

## Supplementary Materials

The supporting information can be downloaded at: https://www.mdpi.com/article/10.3390/ijms241310797/s1.
