# Supplementary material for: Accelerated Bone Loss in Transgenic Mice Expressing Constitutively Active TGF-β Receptor Type I
Source: Int J Mol Sci. 2023 Jun 28;24(13):10797. doi: 10.3390/ijms241310797 (PMC10341885; doi:10.3390/ijms241310797)
Supplement: Supplementary file 1 [file ijms-24-10797-s001.zip › ijms-2364021-supplementary.pdf]

Table S1. Oligonucleotide primers used for qPCR analysis.

Gene	Forward sequences (5’ to 3’)	Reverse sequences (5’ to 3’)
Tnfsf11 (RANKL)	CAAGCTCCGAGCTGGTGAAG	CCTGAACTTTGAAAGCCCCA
Tnfrsf11b (OPG)	AAGAGCAAACCTTCCAGCTGC	CACGCTGCTTTCACAGAGGTC
Ctsk	AGGCATTGACTCTGAAGATGCT	TCCCCACAGGAATCTCTCTG
Sufu	GTGCTATTGCCTTCCAAGCG	GCAGTGGGCTTTACCCTCTT
Ifng (IFN)	AAATCCTGCAGAGCCAGATTAT	GCTGTTGCTGAAGAAGGTAGTA
Acp5 (TRAP)	GATCCCTCTGTGCGACATCA	CCAGGGAGTCCTCAGATCCA
Tnf (TNF-α)	TTGTCTACTCCCAGGTTCTCT	GAGGTTGACTTTCTCCTGGTATG
Ccl2 (MCP-1)	TTGTCTCTATGGCTGCTGTTC	GACTCAAAGTGACCTGCTTCT
Csf1r (c-Fms)	TGGCATCTGGCTTAAGGTGAA	GAATCCGCACCAGCTTGCTA
Csf1 (M-CSF)	ACCTGTTTCCCAAGAAGAGAGCCT	AGCTGTCAACACAAGCAGCCAAAG
Gli1	ACCCGGGATACAACCCAAAT	AGACCATTGCCCATCACAGA
Wnt3a	TGGCAGAATGAGGCATGGAG	TCAGGAAAGCTCTGCCAAGG
Fgf23	AGGACCAGCTATCACCTACA	CGAGTCATGGCTCCTGTTATC
Runx2	GTCATGGCCGGGAATGATGA	CTGCCTGGGATCTCGTCCG
Sp7 (Osx)	CCCTTCTCAAGCACCAATGG	AAGGGTGGGTAGTCATTTGCATA
Alpl (ALP)	CTTGACTGTGGTTACTGCTGATCA	GTATCCACCGAATGTGAAAACGT
Ibsp (Bsp)	TGGCGACACTTACCGAGCTT	CCATGCCCCTTGTAGTAGCTGTA
Col1a1 (Type I collagen)	CCCAAGGAAAAGAAGCACGTC	ACATTAGGCGCAGGAAGGTCA
Bglap (Ocn)	GCTGCCCTAAAGCCAAACTCT	AGAGGACAGGGAGGATCAAGTTC
Ptch1	AGGCGCTAATGTTCTGACCA	CCTCCTGCCAATGCATATAC
Ptch2	TGGCTTCTCCCACAAGTTCA	CAGGAAGGTGCTCTGCAAGG
Shh	TCCGATGTGTTCCGTTACCA	CCTGAGGACTTGTGAGCTGT
Tgfb1	GGTGGTATACTGAGACACCTTG	CCCAAGGAAAGGTAGGTGATAG
TβRI	CGTTGGGTCTTCTCACTGCT	TAACAGAGCCCAGCTGCTTC
TβRII	TTTCCTGTTTCCCTCTCGGC	TCACTGTTAACCGACTTGGGA
Gapdh	TGCACCACCAACTGCTTAG	GGATGCAGGGATGATGTTC
